# DDA: a traceable multi-agent framework for automated structure-based drug design

**DOI:** 10.3389/fchem.2026.1914886

**Published:** 2026-09-01

**Authors:** Yutao Yu, Sicheng Tian, Guohua Wang, Yang Li

**Affiliations:** 1 College of Computer Science and Artificial Intelligence, Northeast Forestry University, Harbin, China; 2 Faculty of Computing, Harbin Institute of Technology, Harbin, China

**Keywords:** automated drug discovery, multi-agent systems, large language models, molecular generation, bioinformatics

## Abstract

Structure-Based Drug Design (SBDD) is a key computational paradigm that uses protein structural information to design and optimize small molecules with desired binding properties. Existing SBDD methods mainly focus on molecular design and often lack the capability to independently conduct downstream evaluation and optimize workflows. Meanwhile, directly applying large language models (LLMs) to molecular design faces challenges such as fragmented execution, limited tool coordination, and insufficient traceability of intermediate decision-making processes. To address these limitations, we propose a Drug Discovery Agent (DDA), a traceable and auditable multi-agent biomedical informatics framework for automating SBDD. DDA uses a role-specific multi-agent architecture to transform natural-language drug-discovery goals into actionable scientific workflows. Through a unified tool-calling protocol, this framework seamlessly integrates bioinformatics, molecular modeling, and molecular docking modules, enabling autonomous workflow execution from target preparation and molecular generation to multi-objective evaluation, candidate prioritization, and trajectory tracking. We systematically evaluated DDA on the CrossDocked2020 benchmark. Under the closed-loop delivery protocol, the framework produced 2,000 final candidate records, with a joint screen-pass rate 20 percentage points higher than that of the strongest specialized baseline. These results indicate that DDA provides a scalable, executable, and traceable computational framework that reduces manual coordination in structure-based automated drug discovery and delivers prioritized candidate sets with minimal human intervention.

## 1 Introduction

Structure-based drug design (SBDD) has become a central paradigm in modern drug discovery, leveraging three-dimensional protein structures to guide the design and optimization of bioactive small molecules. Given a disease-associated binding pocket, an SBDD system seeks to generate candidate molecules with favorable binding properties and evaluate their drug-likeness, synthetic accessibility, and biological potential. Automating this process can accelerate early-stage lead discovery, reduce screening costs, and improve the reproducibility and scalability of molecular design. However, successful drug discovery requires more than molecular generation alone. Candidate molecules must undergo a sequence of downstream analyses, including molecular standardization, property prediction, docking-based evaluation, and candidate prioritization. Consequently, automated SBDD demands both effective molecular design and reliable execution of complex computational workflows.

Recent advances in protein structure prediction, geometric deep learning, and molecular generation have substantially expanded the technical foundation of automated SBDD. AlphaFold has dramatically increased the availability of high-quality protein structures for structure-guided drug discovery (Jumper et al., 2021), while benchmark datasets such as CrossDocked2020 provide standardized environments for developing and evaluating structure-based molecular design methods (Francoeur et al., 2020). In parallel, pocket-conditioned and diffusion-based generative models have achieved remarkable progress in structure-aware molecular design by incorporating binding-site geometry and protein–ligand interactions into the generation process (Guan et al., 2023; Peng et al., 2022; Schneuing et al., 2024). More recent approaches further improve molecular quality and target specificity through decomposition strategies, target-aware molecular language models, and guided generation mechanisms (Guan et al., 2024; Wu K. et al., 2024). Despite these advances, most existing methods focus primarily on molecular generation and treat downstream evaluation as an external process. As a result, critical stages of the SBDD pipeline including target preparation, molecular standardization, ADMET assessment, docking validation, candidate prioritization, and result inspection—remain fragmented across heterogeneous tools and often require substantial manual intervention.

This limitation becomes particularly pronounced when considering end-to-end drug discovery workflows. Traditional SBDD campaigns require researchers to coordinate multiple software platforms, file formats, parameter settings, and intermediate outputs, resulting in labor-intensive and difficult-to-reproduce workflows. Meanwhile, recent advances in large language model (LLM) agents have demonstrated strong capabilities in task decomposition, tool utilization, and long-horizon reasoning (Schick et al., 2023; Yao et al., 2022). Multi-agent systems further extend these capabilities through collaborative planning and coordinated tool execution among specialized agents (Wu K et al., 2024), while domain-specific frameworks such as ChemCrow highlight the potential of agent-based systems for automating chemical reasoning and computational experimentation (Bran et al., 2024). Nevertheless, generic agent frameworks are not designed for the stringent requirements of SBDD and remain vulnerable to tool-selection errors, parameter inconsistencies, execution failures, and loss of intermediate workflow states. More importantly, they often lack mechanisms for maintaining structured state transitions and preserving complete provenance throughout the discovery process.

Beyond computational benchmarking, the framework must also support the downstream transition from *in silico* predictions to experimental testing. Its practical utility ultimately depends on its ability to support real-world drug discovery pipelines. Structure-based virtual screening and fragment-based approaches have demonstrated their value in identifying novel chemotypes and optimizing binding affinity (Xu et al., 2021; Zhu et al., 2025), while rational design principles remain essential for guiding lead compound selection and optimization (He et al., 2019; Wang et al., 2019; Ye et al., 2022). Target-aware design strategies continue to expand the accessible chemical space for disease-relevant targets such as kinases, metabolic enzymes, and transporters (Liu et al., 2023; Qin et al., 2024; Zhou et al., 2023). DDA is positioned to bridge the gap between *in silico* candidate generation and experimental testing by providing an executable and auditable workflow that connects computational predictions with downstream prioritization. The framework is immediately applicable to target-focused virtual screening campaigns and lead identification efforts; however, prospective synthesis and biochemical assays remain essential to validate predicted candidates and establish their translational potential (Qin et al., 2024; Ye et al., 2022).

**FIGURE 1:**
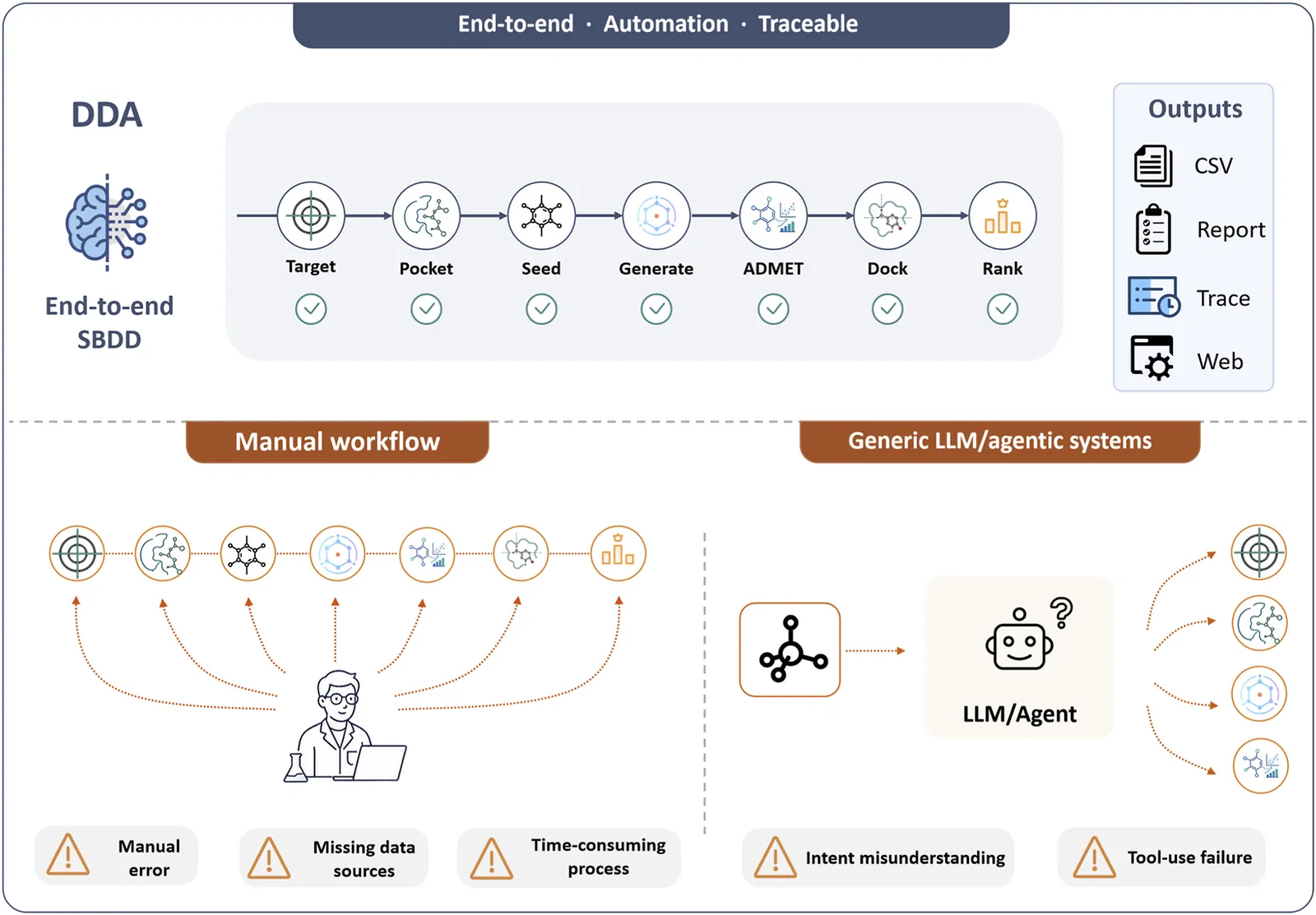
Overview of the workflow-level gap addressed by DDA in automated SBDD. DDA connects target, pocket, seed, generation, ADMET, docking, and ranking into an executable and traceable workflow with CSV files, reports, execution traces, and web-based 2D/3D visualization outputs. The lower part contrasts manual workflows and generic LLM-based agents, which may suffer from human error, missing data sources, time-consuming operation, intent misunderstanding, and tool-use failure.

To address these challenges and bridge the gap between computation and experimentation, we present Drug Discovery Agent (DDA), a traceable multi-agent biomedical informatics framework for automated SBDD. DDA transforms natural-language drug discovery objectives into executable scientific workflows through a role-specialized multi-agent architecture and a unified tool protocol. By orchestrating bioinformatics, molecular modeling, and docking tools within a structured execution environment, DDA enables autonomous workflow execution spanning target preparation, molecular design, multi-objective evaluation, candidate prioritization, and provenance tracking. As illustrated in Figure 1, DDA bridges the gap between isolated molecular generation models and executable, auditable, and fully traceable structure-based drug discovery workflows. To support user interaction and auditability, DDA also provides a web application and a command-line interface that expose task configuration, workflow progress, intermediate products, candidate tables, and molecular visualizations. Users can submit tasks, monitor execution, inspect computational evidence, and review prioritized candidates through these interfaces, enabling closed-loop workflow delivery. The Web interface serves as the primary user-facing execution surface, where researchers can submit drug-design requests, monitor workflow progress, and inspect intermediate results without interacting with the underlying code or command-line tools. This design lowers the barrier for experimental deployment and supports human-in-the-loop review of candidate molecules.

We evaluated DDA on the CrossDocked2020 benchmark and compared it with specialized SBDD generation methods and general-purpose LLM agents. Experimental results show that DDA can stably complete the full end-to-end process from target preparation to candidate prioritization, automatically handling tool calls, file format conversions, and abnormal recovery from intermediate steps. Since the framework prioritizes ensuring that outputs meet the user-specified number of molecules and tends to retain candidates with reasonable physicochemical properties, supplementary generation or multiple screenings are automatically triggered when necessary. This results in a multi-objective success rate about 20 percentage points higher than the specialized generation baseline. However, this improvement mainly comes from framework-level completion and filtering strategies rather than performance breakthroughs in the underlying generation model itself. Compared to the general LLM baseline, DDA retains a more complete intermediate output within the same downstream evaluation toolchain, reflecting its advantages in state management and error recovery. Ablation experiments further confirmed the critical role of planning, tool selection, validation and optimization, and memory I/O for reliably executing long-term workflows. Overall, the main contribution of DDA lies in providing a framework that can replace manual coordination of various discrete tools, automate the handling of repetitive operations and anomalous scenarios, and offer a practical pathway from isolated molecule generation to executable, verifiable, and traceable automated drug design workflows.

## 2 Related work

To position DDA within automated structure-based drug design, we review existing studies from four interconnected directions. Structure-based molecular generation focuses on generating candidate molecules under protein-pocket constraints. Molecular evaluation, ADMET prediction, and docking focus on assessing whether generated candidates are suitable for downstream use. LLM-based agents for biomedical workflows focus on using language models to organize complex biomedical tasks. Automated scientific workflow systems focus on the executability and reproducibility of multi-step scientific computation. These directions respectively address generation, evaluation, intelligent collaboration, and workflow management in SBDD pipelines. Few published systems have considered these components together under a unified structure-conditioned end-to-end execution framework with explicit tool protocols, intermediate products, user-facing execution visibility, and auditable delivery records. Our goal is therefore not to replace specialized generators, but to organize these components within a task-conditioned SBDD workflow framework.

### 2.1 Structure-based molecular generation

The core objective of structure-based molecular generation is to generate candidate ligands under the three-dimensional constraints of protein binding pockets. Unlike target-free molecular generation, SBDD requires generative models not only to produce chemically valid molecules but also to exploit pocket geometry, local atomic environments, and potential interaction patterns so that the generated molecules can match specific target structures. Early pocket-conditioned methods used the protein pocket as spatial context to guide stepwise molecular sampling near the binding site. Pocket2Mol is representative of this direction, as it incrementally generates candidate molecules from 3D protein pockets and thereby couples molecular sampling with pocket geometry (Peng et al., 2022).

As three-dimensional generation tasks impose stronger requirements on geometric consistency, local sampling alone is insufficient to guarantee stability under rotation and translation transformations. Therefore, equivariant neural networks and diffusion models have been introduced into structure-based molecular generation. DiffSBDD and TargetDiff combine SE (3)-equivariant geometry with diffusion-based generation, allowing models to more naturally represent atomic coordinates, local conformations, and pocket constraints in 3D space (Guan et al., 2023; Schneuing et al., 2024). This line of work has advanced structure-based molecular generation from simple pocket-conditioned sampling toward geometry-aware continuous generation. Nevertheless, these methods mainly focus on molecular generation itself and provide limited support for tool-based post-processing, property prediction, task-level candidate ranking, and delivery of validated final products.

Subsequent studies have further incorporated molecular priors and interaction information. DecompDiff decomposes molecules into scaffold and arm regions and improves structure-based generation through decomposed priors (Guan et al., 2024). TamGen introduces target information into chemical language modeling and improves target-aware molecular generation (Wu Q et al., 2024). DiffGui, DiffInt, PoLiGenX, MolCRAFT, and AUTODIFF further introduce property guidance, hydrogen-bond interaction guidance, latent conditioning, continuous-space generation, and autoregressive diffusion to improve local interactions, geometric plausibility, and drug-like properties (Hu et al., 2025; Le et al., 2025; Li et al., 2024; Qu et al., 2024; Sako et al., 2024). In addition to structure-conditioned generation, recent generative AI studies have begun to connect molecular design with synthetic accessibility and experimental validation, showing the importance of moving from computational generation toward practically verifiable drug-design outputs (Swanson et al., 2024a). These studies indicate that structure-conditioned generative models are evolving from answering whether pocket-related molecules can be generated to addressing how more reasonable, optimizable, synthesizable, and drug-design-oriented candidates can be generated.

Despite these advances, most structure-based generative models still mainly serve the molecular sampling stage. Real SBDD tasks require additional steps, including target preparation, structural-context definition, seed construction, molecular standardization, ADMET prediction, docking evaluation, multi-objective ranking, result presentation, and inspection of intermediate products. Generative models usually do not automatically manage input-output files, tool invocation protocols, failure states, or delivery status. Therefore, our work does not aim to replace specialized generators. Instead, DDA incorporates structure-conditioned molecular generators as pluggable tools within an end-to-end execution framework, enabling molecular generation to form a continuous workflow with downstream ADMET prediction, docking, ranking, recovery-aware validation, and user-facing auditability. This design transforms generative models from isolated sampling modules into reliable components of executable SBDD workflows, which is also consistent with the view that machine learning-aided generative molecular design should integrate property evaluation, synthetic feasibility, and downstream validation (Du et al., 2024).

### 2.2 Molecular evaluation, ADMET prediction, and docking

After candidate molecules are generated, determining whether they have downstream development value is equally critical in SBDD. A molecule with a favorable predicted binding score may still be unsuitable for subsequent studies due to poor drug-likeness, synthetic difficulty, ADMET risks, or irreproducible results. Therefore, modern molecular design usually needs to jointly consider drug-likeness, synthetic accessibility, physicochemical properties, ADMET-related properties, and docking-oriented scores. QED provides a quantitative drug-likeness metric based on multiple desirability functions (Bickerton et al., 2012). The synthetic accessibility score estimates molecular synthetic difficulty (Ertl and Schuffenhauer, 2009). Lipinski’s rule-of-five remains a classical heuristic for early screening of oral drug-like properties (Lipinski et al., 1997). These metrics are computationally efficient and suitable for preliminary filtering and ranking of large candidate sets.

As generative models can output larger candidate molecule collections, relying only on basic physicochemical properties becomes insufficient for drug-design decision-making. ADMET prediction extends the scope of molecular evaluation and enables systems to preliminarily assess pharmacokinetic and toxicity risks before experimental validation. ADMET-AI provides a machine-learning platform for evaluating large-scale chemical libraries (Swanson et al., 2024b). ADMETlab 3.0 further expands online ADMET prediction in endpoint coverage, model performance, decision support, and API functionality (Fu et al., 2024). These tools improve the automation of candidate screening, but they also impose higher requirements on molecular input formats, standardization procedures, field mapping, and result alignment. If SMILES strings cannot be parsed, canonicalization is inconsistent, or key fields are missing from result tables, ADMET outputs cannot be reliably used for candidate ranking.

Molecular docking remains a core evaluation step in structure-based drug design. AutoDock Vina has been widely adopted in virtual screening due to its efficient scoring function and optimization strategy (Trott and Olson, 2010). Uni-Dock further accelerates Vina-family docking using GPUs and enables more efficient large-scale docking and virtual screening (Yu et al., 2023). However, docking scores are affected by the docking engine, scoring function, pocket definition, conformer preparation, and parameter settings, therefore cannot be directly interpreted as experimental binding affinities. Automated SBDD should not optimize docking scores alone. Instead, docking scores should be used as docking-oriented computational signals and jointly considered with ADMET, QED, SA, and other evaluation signals for candidate ranking.

Although these evaluation tools are mature, their integration into automated workflows remains challenging. Molecular generation, standardization, ADMET prediction, docking evaluation, and final ranking are separated by multiple schema boundaries and file boundaries. Once intermediate states are missing or output formats are inconsistent, downstream evaluation can fail. In DDA, we organize RDKit (Landrum, 2006), pandas (McKinney, 2010), ADMET-AI, and Uni-Dock/Vina-family docking into a unified downstream evaluation pipeline. Through the Tool Registry, GlobalMemory, recorded execution products, and user-facing result inspection, DDA records inputs, outputs, failure states, and ranking criteria. As a result, evaluation modules are no longer isolated tools invoked independently, but together form an inspectable, reproducible, and traceable candidate-screening chain. The resulting values remain computational prioritization signals rather than experimental measures of affinity, efficacy, or clinical suitability.

### 2.3 LLM-based agents for biomedical workflows

Large language models and LLM-based agents provide a new technical path for automating complex biomedical workflows. Foundation models are changing the organization of data analysis, sequence modeling, structure prediction, and tool-based workflows in bioinformatics (Guo et al., 2025). LLM-based agents further extend these capabilities to natural-language task understanding, tool selection, and workflow organization. ReAct shows that language models can alternate between reasoning and acting in external environments, thereby combining language reasoning with tool use (Yao et al., 2022). Toolformer further demonstrates that language models can learn to use external tools through API-like calls (Schick et al., 2023). AutoGen extends multi-agent conversations to complex application scenarios such as tool invocation, code execution, and human-agent collaboration (Wu Q. et al., 2024). Related surveys also indicate that planning, memory, tool use, reflection, and multi-agent collaboration are key capabilities of LLM-based autonomous agents (Xi et al., 2025). These studies provide the foundation for applying LLM agents to complex scientific tasks.

In biomedical informatics, LLM-based agents have begun to support medical decision-making, medical image analysis, and biological data analysis. MDAgents uses adaptive collaboration among LLM agents to support medical decision-making (Kim et al., 2024). MedSegAgent maps natural-language segmentation requests to appropriate datasets, labels, and segmentation models, thereby constructing a universal multi-agent system for instructive medical image segmentation (Huang et al., 2026). CellAgent uses an LLM-driven multi-agent framework to automate single-cell data analysis workflows (Xiao et al., 2024). These studies show that LLM agents can serve as a coordination layer between natural-language requirements and professional biomedical computing tools, especially in task planning, tool selection, and result interpretation.

Drug discovery has also begun to explore agentic workflows. DrugAgent automates AI-aided drug discovery programming through LLM multi-agent collaboration, focusing on data acquisition, model construction, code generation, and evaluation (Liu et al., 2024). DrugMCTS combines multi-agent collaboration, retrieval-augmented generation, and Monte Carlo Tree Search for structured reasoning and search in drug repurposing (Yang et al., 2025). ChemCrow provides a tool-augmented chemistry-agent paradigm for chemical reasoning and computational experimentation (Bran et al., 2024). Meanwhile, general-purpose LLMs represented by Llama three have further promoted the exploration of LLMs in complex scientific tasks (Grattafiori et al., 2024). These studies advance the use of LLM agents in drug discovery, but they focus more on general programming, chemistry tool use, reasoning, or drug repurposing rather than the specific local structure-conditioned SBDD workflow considered here.

ChemCrow is particularly relevant to the positioning of DDA because it demonstrates the value of combining LLM reasoning with expert chemistry tools. DDA shares this general tool-augmented-agent principle, but extends it toward a structure-conditioned SBDD delivery setting in which local target inputs, structural-context preparation, molecular generation, downstream candidate evaluation, final candidate selection, and docking-score write-back must be coordinated as one workflow. The distinction is not merely the use of different molecular tools. DDA additionally organizes registry-grounded tool selection, task-specific executable wrappers, code evaluation and bounded refinement, explicit runtime state, artifact inspection, bounded delivery recovery, and Web/CLI audit visibility. Table 1 summarizes this capability-level positioning in a qualitative framework comparison. It is not intended as a head-to-head molecular-quality evaluation.

**TABLE 1:** Capability-level positioning of ChemCrow and DDA.

Aspect	ChemCrow	DDA
Scope	General chemistry reasoning, synthesis planning, literature/database interaction, and chemistry-tool use	Structure-conditioned molecular-design workflow delivery from local target input to ranked candidate products
Tool use	LLM-mediated use of chemistry-oriented expert tools	Planner, registry-grounded tool selection, executable-wrapper generation, evaluation/refinement, deterministic execution, and shared runtime state
Structural context	No matched local CrossDocked/TamGen structural workflow in the compared public framework	Local protein preparation, reference-ligand or user-provided structural context, and center-guided workflow configuration
Generation	General chemistry-agent tool scope	Target-aware TamGen generation integrated with structural preparation and downstream processing
Prioritization	General chemical reasoning and tool interaction	ADMET-oriented prioritization, Top- N selection, final-candidate docking, and score write-back
Product management	General tool outputs and agent interaction records	GlobalMemory, output detection, product validation, explicit partial status, and bounded quantity recovery
User interface	Programmatic or chat-style chemistry-agent interaction	Web application and CLI with task configuration, execution status, logs, result tables, molecular visualization, and audit visibility

This comparison clarifies both the relationship and the methodological contribution of DDA. ChemCrow establishes an important general chemistry-agent paradigm, whereas DDA focuses on a constrained, state-aware workflow layer for local SBDD toolchains. The present study therefore compares DDA with specialized structure-based molecular generators when evaluating molecular-output properties, while using ChemCrow to position the framework-level contribution. A future same-tool comparison with a generic ReAct/LangChain-style controller will be important for isolating the contribution of workflow architecture from differences in tools, structural representations, and downstream delivery policies. Note that ChemCrow and DDA do not currently share an identical tool registry, structural input representation, molecular generator, output contract, or runtime budget. Such a matched comparison would require a common generic controller supplied with the same tool set and constraints.

SBDD imposes more specific reliability requirements on LLM agents. The system must not only understand natural-language design goals but also correctly identify target structures, structural-context information, molecular inputs, tool parameters, output schemas, and downstream evaluation dependencies. General LLMs or agentic systems can be prone to intent misunderstanding and tool-use failure, including incorrectly interpreting task goals, invoking nonexistent tools, generating incompatible parameters, failing to parse tool outputs, or failing to pass intermediate results to downstream steps. Coordinated AI agents in healthcare and biomedical scenarios require explicit role division, collaboration mechanisms, and verification controls (Moritz et al., 2025), which is highly consistent with the execution requirements of SBDD toolchains. Therefore, in DDA, we constrain the role of LLM agents to task interpretation, workflow-step instantiation, registry-grounded tool selection, code-wrapper generation, and verification support, rather than allowing them to directly replace professional molecular generators or docking tools. The scientific workflow scaffold, tool interfaces, output paths, and validation constraints are defined by domain knowledge. Deterministic modules execute scientific backends, validate products, and maintain runtime state. Through this design, DDA preserves the natural-language adaptability of LLM agents while constraining their behavior using tool protocols, execution verification, and structured state management, making them suitable for auditable SBDD automation.

### 2.4 Automated scientific workflow systems

Automated scientific workflow systems have long been used in computational biology and biomedical data analysis. Their core value lies in improving reproducibility, scalability, and execution management. Snakemake provides a Python-based workflow language for building reproducible and scalable bioinformatics pipelines (Köster and Rahmann, 2012). Nextflow supports portable workflow execution across local, cluster, and cloud environments and has been widely used in large-scale data analysis (Di Tommaso et al., 2017). These workflow engines are effective at managing task dependencies when workflow structures, input-output files, and execution environments are predefined, thereby significantly improving the stability of complex computational pipelines.

However, traditional workflow engines usually require users to predefine task graphs, parameters, and scripts. When scientific goals change, input structures are incomplete, reference ligand availability varies, or tool selection depends on task context, the flexibility of fixed workflows becomes limited. LLM-based agents provide a natural-language interface for task interpretation and workflow adaptation. Recent multi-agent systems for scientific discovery further show that specialized agents can collaborate across literature retrieval, hypothesis generation, experimental suggestion, data analysis, and result interpretation, thereby supporting more automated scientific discovery workflows (Ghareeb et al., 2026). Automated scientific discovery is also moving from equation discovery toward more complex autonomous discovery systems (Kramer et al., 2026). These trends indicate that future scientific workflows need automation, task adaptation, and process interpretability at the same time.

However, in executable drug-design scenarios, agentic flexibility alone remains insufficient. Unconstrained code generation may lead to hallucinated APIs, invalid imports, incorrect file paths, missing outputs, and runtime failures. Conversely, traditional workflow engines alone are difficult to adapt workflow paths according to natural-language goals, target structures, and intermediate products without additional task-specific logic. The blackboard problem-solving architecture provides a classical idea for coordinating multiple modules through a shared workspace (Nii, 1986), which directly inspires state transfer in complex scientific workflows. Therefore, automated SBDD requires a hybrid design that combines the task understanding and in-context planning capabilities of LLM agents with the tool constraints, state management, deterministic execution, product validation, and user-auditable execution surfaces of workflow systems.

DDA is designed to address this workflow-level gap. Compared with traditional workflow engines, DDA instantiates and adapts supported SBDD workflow paths within a domain-expert-defined scaffold according to natural-language requests, available products, and documented tool constraints. Compared with general LLM agents, DDA constrains each execution step through documented tool protocols, structured state transfer, pre-execution verification, runtime artifact recording, bounded recovery, and explicit delivery validation. This allows DDA to maintain task-conditioned flexibility while supporting inspectable and reproducible workflow delivery. For automated drug design, the significance of this hybrid workflow-agent design is that it advances scientific tasks from being merely conversational to being executable, verifiable, user-auditable, and suitable for subsequent computational and experimental decision making.

## 3 Methodology

### 3.1 Problem definition

Given a target protein structure or protein identifier P , optional reference-ligand or structural-context information $L_{r}$ , and a natural-language drug-design request q , the goal of automated structure-based drug design is to generate a ranked set of candidate molecules together with supporting property predictions, docking-oriented evaluations, recorded workflow products, and visualization outputs. The output of the system is formulated in Equation 1 as:

$$Y=\left\{C_{\text{rank}},A_{\text{admet}},D_{\text{dock}},T_{\text{trace}},V_{\text{vis}}\right\}.$$ (1)

Here, $C_{\text{rank}}$ denotes the ranked candidate molecule set, $A_{\text{admet}}$ denotes ADMET prediction results, $D_{\text{dock}}$ denotes docking-oriented evaluation results, $T_{\text{trace}}$ denotes execution traces and intermediate file records, and $V_{\text{vis}}$ denotes front-end visualization results. These outputs provide computational prioritization evidence for downstream drug-candidate discovery, filter out compounds that are competitive across multiple metrics through artificial intelligence, and offer an end-to-end automated generation pipeline to support subsequent experimental validation, thereby substantially improving research efficiency.

An automated SBDD workflow should satisfy three basic requirements. First, stage coverage requires the system to cover the scientific stages needed by the current task, rather than only completing molecular generation. Second, stage ordering requires upstream products to be produced before downstream steps are invoked. For example, structural-context information and seed molecules should be prepared before structure-conditioned molecular generation, and standardized candidate molecules should be obtained before ADMET prediction and docking evaluation. Third, execution consistency requires each step to use documented tools, valid parameters, valid paths, and parseable output schemas, thereby reducing cases in which a tool appears to run successfully but its output cannot be used by subsequent stages.

Automated SBDD is therefore not a single molecular-generation task, but a scientific workflow problem involving multi-stage tool execution, state transfer, product validation, result aggregation, and user-facing inspection. The methodology addresses three central questions: how to instantiate an ordered workflow from a natural-language request within a domain-expert-defined scientific scaffold; how to ensure that each tool invocation follows an executable input-output protocol; and how to preserve intermediate states and recorded evidence across multiple steps.

### 3.2 Overview of DDA

To satisfy stage coverage, ordered dependencies, and execution consistency, we design DDA as a collaborative multi-agent framework for automated SBDD. DDA transforms a natural-language request q , a target protein P , and optional structural-context information $L_{r}$ into an executable workflow, as defined in Equation 2:

$$W=\left(s_{1},s_{2},\ldots ,s_{T}\right).$$ (2)

Here, $s_{t}$ denotes the t -th workflow step. Each step contains the current task objective, required inputs, available tools, expected outputs, and validation conditions. The scientific workflow scaffold, stage dependencies, tool interfaces, output paths, and validation constraints are specified by domain knowledge. DDA does not rely on an LLM to derive molecular-design principles or scientific procedures from scratch. Instead, the LLM assumes a bounded, operational role: it interprets user requests, defines workflow steps according to the task context, selects tools from the registry, and generates executable wrappers. All scientific computations, along with product validation, state maintenance, and result recording, are handled by deterministic tools and runtime controllers operating under explicit tool protocols and shared-state constraints.

**FIGURE 2:**
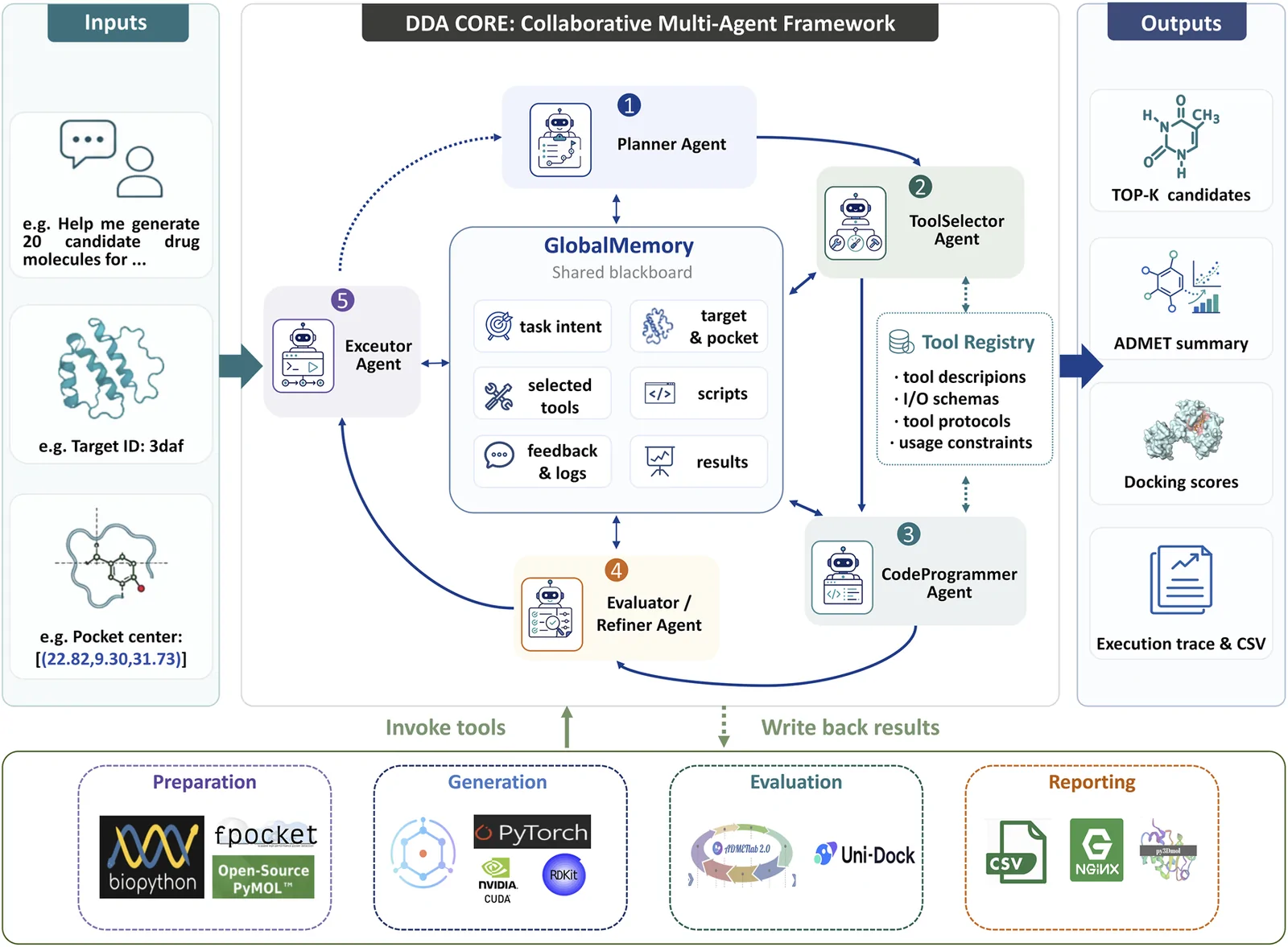
DDA collaborative multi-agent architecture. The collaborative multi-agent architecture of DDA, showing how the system transforms a user request and target structure into an end-to-end SBDD workflow through agent collaboration, tool-grounded execution, and shared-state management.

Figure 2 illustrates the overall architecture of DDA. The system inputs include a natural-language request, a target structure or target identifier, and optional structural-context information. The DDA core consists of the Planner Agent, ToolSelector Agent, CodeProgrammer Agent, Evaluator/Refiner Agent, Executor Agent, and GlobalMemory. The Planner Agent instantiates ordered workflow steps from the user goal, available products, and the canonical SBDD scaffold. The ToolSelector Agent selects relevant tools according to the current step. The CodeProgrammer Agent generates executable code blocks required by the current step. The Evaluator/Refiner Agent validates each code block against the declared tool protocol, expected file paths, and output-contract consistency prior to execution. Any detected inconsistency triggers a bounded revision loop. The Executor Agent runs code blocks in a controlled manner and records runtime results. GlobalMemory stores task intent, target and structural-context information, selected tools, code blocks, feedback logs, execution status, and intermediate products across steps.

At the functional level, DDA organizes SBDD into six connected stages. Target preparation reads and standardizes the target structure, providing the structural basis for downstream pocket localization and docking evaluation. Structural-context and seed setup determines a reference-ligand-derived or user-provided center and prepares seed molecules, providing spatial constraints and initial molecular information for structure-conditioned generation. Molecular generation produces candidate molecules based on the structural context and seed information. Molecular processing parses, standardizes, deduplicates, and tabulates generated molecules so that downstream evaluation tools can read them. Property and docking evaluation produces ADMET predictions and docking-oriented scores based on standardized candidates. Reporting and visualization merge candidate molecules, evaluation results, execution records, and front-end rendering outputs into inspectable results. Through these connected stages, DDA instantiates a task-conditioned executable chain from input to result.

Traditional fixed workflows apply the same full pipeline to every task, regardless of the user’s actual request. DDA, in contrast, adapts its execution path to the specific task: for example, it can run only ADMET evaluation on existing molecules, perform docking on previously generated candidates, or resume an incomplete run from its last valid checkpoint. The LLM’s role is therefore not to invent new scientific protocols from scratch, but to select and configure appropriate workflow paths from a domain-defined set, while the deterministic runtime manages state, recovery, and output validation.

### 3.3 Tool registry and tool-grounded execution

Automated SBDD depends on heterogeneous tools, which often have different input formats, output fields, parameter constraints, and runtime environments. If agents freely generate invocation logic only from natural-language descriptions, they may produce nonexistent tool interfaces, incorrect parameters, invalid file paths, or unparseable outputs. DDA therefore uses a Tool Registry to constrain tool invocation, ensuring that each tool call is grounded in explicit protocols.

The Tool Registry is denoted as R . Each entry records a tool name, task role, supported input products, expected output products, invocation protocol, and usage constraints. The registry also provides the contextual information needed for executable-wrapper generation and product validation. Detailed registry entries and invocation protocols are provided in the framework implementation and reproducibility package. For workflow step $s_{t}$ , the ToolSelector Agent selects a tool subset according to the current step, the Tool Registry, and the previous shared state, as defined in Equation 3:

$$U_{t}=ToolSelector\left(s_{t},R,B_{t-1}\right).$$ (3)

Here, $U_{t}$ denotes the tool set required by step $s_{t}$ , and $B_{t-1}$ denotes the shared state before executing step $s_{t}$ . By exposing only the tools and protocols required by the current step, DDA reduces selection interference caused by excessive tool context and decreases the risk of tool-invocation and output-schema inconsistency.

Table 2 summarizes the major tool groups and input-output protocols integrated in DDA. The tool groups cover target preparation, structural-context and seed setup, molecular generation, molecular processing, property and docking evaluation, and result aggregation and front-end visualization. The Tool Registry is extensible: a new tool can be added by defining its supported task role, input and output products, invocation constraints, and validation expectations. This design enables users to extend the workflow with compatible generators, property predictors, docking engines, synthesis-planning services, or experimental interfaces while preserving explicit input-output contracts.

**TABLE 2:** Tool groups and input-output protocols integrated in DDA.

Tool group	Representative tools	Inputs	Outputs
Target preparation	BioPython; uni-dock utilities	PDB ID; PDB/CIF files; target metadata	Prepared receptor; target metadata
Structural-context and seed setup	RDKit; seed construction scripts	Target structure; reference ligand; user-provided center; ligand records	Structural center; seed SMILES; pretreatment files
Molecular generation	TamGen pipeline; generation scripts	Seed file; structural context; model checkpoint	Raw molecules; generation outputs
Molecular processing	RDKit; pandas; post-processing scripts	Raw generated files; molecule tables	Canonical SMILES; candidate tables
Property and docking evaluation	ADMET-AI; uni-dock; RDKit descriptors	Candidate SMILES; receptor; docking center/box	ADMET table; docking scores; ranked candidates
Result aggregation and visualization	Pandas; RDKit drawing; 2D/3D viewers; front-end modules	Candidate tables; evaluation outputs; recorded workflow products	Ranked results; 2D/3D views; execution records; CSV files

During execution, tool selection is not performed through one-time global exposure. Each workflow step receives only the tool subset and corresponding protocols relevant to the current task. The purpose is not merely to reduce the number of visible tools, but to reduce interference from irrelevant tool descriptions during code-block generation and execution. As a result, the CodeProgrammer Agent generates executable logic around the required inputs, outputs, and constraints of the current step. For complex SBDD tasks, tool-grounded execution reduces hallucinated APIs, parameter errors, path errors, and result-schema inconsistencies.

### 3.4 Planning and step-wise agent collaboration

A Tool Registry alone cannot guarantee correct workflow execution, because the system must also determine when to invoke each tool and how to use the output of one step as the input of the next step. DDA therefore uses the Planner Agent to translate a natural-language request into a structured workflow. The Planner instantiates and adapts ordered steps within the canonical SBDD scaffold according to the user goal, available products, local input constraints, and task requirements.

Given a natural-language request q , a target protein P , optional structural-context information $L_{r}$ , and an initial shared state $B_{0}$ , the Planner Agent generates the workflow, as defined in Equation 4:

$$W=Planner\left(q,P,L_{r},B_{0}\right).$$ (4)

For a standard full SBDD task, the Planner usually generates a sequence covering target preparation, structural-context and seed setup, molecular generation, molecular processing, ADMET evaluation, docking evaluation, and final reporting. Depending on the request and available products, it can specify whether stages should be reused, executed, validated, aggregated, or recovered. For example, an ADMET-only request with existing molecule tables can bypass molecular generation; a docking-only request can reuse existing final candidates; and a task with explicit center coordinates can use the supplied structural constraint rather than relying on automatic center preparation. Each step not only describes what should be done, but also defines the inputs on which it depends, the outputs that should be generated, and how subsequent steps should read these outputs.

For each step $s_{t}$ , the CodeProgrammer Agent generates an executable code block according to the step description, selected tool set, and shared state, as defined in Equation 5:

$$K_{t}^{\left(0\right)}=CodeProgrammer\left(s_{t},U_{t},B_{t-1}\right).$$ (5)

Here, $K_{t}^{\left(0\right)}$ denotes the initial code block. The code block refers to executable tool-invocation logic for the current step. It may include local tool calls, command protocols, input-output organization, and result-parsing logic, but it is not restricted to a specific programming language or script form.

The code block is not executed immediately. Instead, it is first checked by the Evaluator/Refiner Agent. The Evaluator/Refiner verifies whether the code block satisfies the declared task objective, tool protocol, input path, output path, result schema, and task constraints of the current step. This validation checks operational and product-contract consistency. If the code block does not pass verification, the Refiner generates a revised version according to the feedback, as defined in Equation 6:

$$K_{t}^{\left(r+1\right)}=Re\mathrm{fi}ner\left(K_{t}^{\left(r\right)},F_{t}^{\left(r\right)},s_{t},U_{t},B_{t-1}\right).$$ (6)

Here, $F_{t}^{\left(r\right)}$ denotes the verification feedback at the r -th refinement round. To avoid unbounded repair, DDA uses a bounded code-level refinement policy. After a code block passes verification or reaches the permitted refinement condition, the Executor Agent executes the current code block and records runtime status, output paths, error messages, return codes, and result products. This step-wise collaboration enables DDA to perform task interpretation, tool grounding, execution verification, and result writing at each stage, rather than relying on one-shot generation of an entire workflow.

### 3.5 GlobalMemory and traceable state transfer

One core risk in long-chain SBDD workflows is intermediate-state loss. Even if a single tool call succeeds, subsequent steps may still fail if output file paths, candidate molecule tables, structural centers, ADMET results, or docking results are not properly recorded. DDA therefore uses GlobalMemory as the shared state-management mechanism of the current workflow, instead of relying on implicit context or manual recording.

GlobalMemory is denoted as $B_{t}$ , representing the shared state after executing step $s_{t}$ . It stores task intent, target and structural-context information, selected tools, generated code blocks, feedback logs, execution status, intermediate products, and result tables. Unlike semantic memory used for long-term historical retrieval, GlobalMemory in DDA mainly serves as a shared blackboard for the current workflow. This design is consistent with the blackboard problem-solving architecture, which coordinates multiple modules through a shared workspace (Nii, 1986).

After step *s*_*t*_ is completed, the system state is updated as defined in Equation 7:

$$B_{t}=Update\left(B_{t-1},O_{t},E_{t},F_{t}\right).$$ (7)

Here, $O_{t}$ denotes the output products generated by the step, $E_{t}$ denotes runtime information, and $F_{t}$ denotes verification feedback and error information. Through this state update, subsequent steps can locate upstream files, check whether expected outputs exist, identify failure types, and continue execution using verified products. GlobalMemory therefore organizes otherwise scattered files, parameters, and results into a continuous workflow state. This enables users and downstream agents to inspect the recorded computational path from generation to evaluation and final candidate output.

### 3.6 Structure-conditioned generation and result output

The key stage of SBDD is to generate candidate molecules from target structures and structural-context information, and then transform these candidates into results that can be evaluated, ranked, and inspected. In this stage, DDA does not merely invoke a molecular generation tool. Instead, it organizes generation, processing, evaluation, and delivery validation into a continuous process so that final results can be reviewed by researchers and used in subsequent computational or experimental decision making.

For target protein *P*, the system determines the structural center according to a reference ligand or user-provided information, as defined in Equation 8:

$$c_{P}=\left(x_{P},y_{P},z_{P}\right).$$ (8)

In the benchmark setting, $c_{P}$ is calculated from the heavy-atom geometric center of the reference ligand in the local test complex. This setting ensures that structural-context definition is reproducible across targets and avoids additional uncertainty introduced by online structure retrieval or de novo pocket prediction. In practical use, DDA also supports explicitly provided center coordinates and ligand-derived structural-context information.

Given target protein P , structural center $c_{P}$ , seed molecule set $S_{P}$ , and generation configuration Θ , DDA invokes the structure-conditioned molecular generation tool to produce raw candidate molecules, as defined in Equation 9:

$$G_{P}=Generate\left(P,c_{P},S_{P},\Theta \right).$$ (9)

Here, $G_{P}$ denotes the set of raw generated molecules. The system then performs parsing, canonicalization, deduplication, and sanitization to obtain the candidate molecules defined in Equation 10, which can enter downstream evaluation:

$$M_{P}=\left\{m_{i}\in G_{P}|Valid\left(m_{i}\right)=1\right\}.$$ (10)

The candidate set $M_{P}$ is then passed to ADMET prediction and docking-oriented evaluation. At this stage, DDA records molecule tables, property-prediction tables, docking-result tables, execution status, and failure flags, and aggregates them into ranked candidates, ADMET summaries, docking-oriented scores, recorded execution products, CSV files, and web visualization outputs. A docking-oriented score is not interpreted as experimental binding affinity. Instead, DDA organizes generated molecules and downstream evaluation results into an inspectable computational evidence chain.

After normal execution produces candidate products, deterministic validators inspect the final candidate table against the requested delivery count, required molecular fields, and docking-oriented score fields. The runtime records the validation outcome together with relevant output paths, execution status, and available intermediate products. This validation process is defined in Equation 11:

$$V=Validate\left(C_{\text{rank}},N_{\text{req}},\Phi \right).$$ (11)

Here, $C_{\text{rank}}$ denotes the ranked candidate molecule set, $N_{\text{req}}$ denotes the user-requested number of final candidates, and Φ denotes the set of required fields that each candidate must contain (e.g., SMILES, QED, SA, ADMET properties, docking score). The validation outcome V is a status variable that records whether the delivery contract is satisfied, and if not, which specific conditions remain unmet.

If the number of final candidates is below $N_{\text{req}}$ , DDA can initiate bounded supplementation. The controller expands the generation budget according to a predefined recovery schedule, repeats molecular processing, ADMET-oriented evaluation, candidate selection, docking-score write-back, and final product validation. Each supplementation event is recorded in a target-level audit file. This process is bounded: if the requested delivery contract remains unmet after the configured retry budget is exhausted, the workflow returns an explicit partial-delivery status rather than silently reporting the task as complete.

The final output contains not only Top- K molecules, but also intermediate products supporting candidate ranking, result verification, and delivery audit. The final result is reported together with the conditions under which the requested candidate-delivery contract was met, reused, recovered, or remained partial.

## 4 Experiments and results

### 4.1 Dataset

We evaluated DDA on a fixed 100-target test set from CrossDocked 2020. CrossDocked2020 is a commonly used benchmark for structure-based drug design and contains cross-docked protein–ligand complexes that support the evaluation of pocket-conditioned molecular generation and docking-oriented candidate prioritization (Francoeur et al., 2020). In our experiments, each target corresponds to a local test complex. DDA receives the target structure, reference-ligand or structural-context information, and a user-specified candidate-delivery request. The system then instantiates the supported workflow path and returns ranked candidate products together with available ADMET-related and docking-oriented fields.

To ensure reproducible structural-context definition, the benchmark uses the heavy-atom geometric center of the corresponding reference ligand in the local test complex. This protocol provides a consistent center for target preparation, local residue selection, and docking configuration across targets. It is a controlled benchmark setting rather than a de novo pocket-discovery experiment, and it does not use Fpocket. In practical use, DDA also supports explicitly provided center coordinates, local structural files, and ligand-derived structural-context information.

### 4.2 Experimental environment

For the production Web and command-line workflow, DDA uses DeepSeek-V4-Flash through the ChatDeepSeek interface. The model identifier, provider endpoint, temperature, and maximum output length are configured through environment variables. The standard operational configuration uses a temperature of 0.1 and a maximum output length of 4,000 tokens. The fallback identifier deepseek-coder in the source code is not the model used for the production workflow; it is activated only when the configured model variable is absent.

To avoid unbounded wrapper repair, the implementation applies a bounded refinement policy: a generated wrapper receives at most two refinement attempts after evaluator feedback. The executor applies step-specific runtime controls and records standard output, standard error, return codes, and output products. API keys, private endpoint credentials, and machine-specific paths are not included in the reproducibility package. The release instead provides an environment template and non-sensitive configuration documentation.

For DDA, the reported 20 candidates per target are final records produced under the closed-loop delivery protocol. After normal workflow execution, deterministic validators inspect the final candidate products against the requested count and required downstream fields. When the delivery contract is not met, bounded supplementation can expand the generation budget, repeat post-processing and ADMET-oriented evaluation, re-select final candidates, perform docking-score write-back, and revalidate the results. This setting is closer to practical drug-design scenarios, where users typically need evaluated and ranked candidate molecules rather than large numbers of unfiltered raw samples.

### 4.3 Baselines

The experiments evaluate two complementary levels of evidence. The first level consists of molecular-pipeline and delivery outcomes. These include the final candidate records produced by DDA, fresh matched raw-20 TamGen controls, specialized SBDD generator outputs, and direct target-informed text-to-SMILES baselines. The second level consists of framework-mechanism evidence from Ablation Analysis, which separately evaluates planning, tool selection, executable wrapper generation, and state-aware recovery on controlled task suites. These levels measure different properties and should not be interpreted as estimates of a single common success rate.

The LLM roles differ across these baselines and are reported separately. The direct text-to-SMILES baselines use GPT-5.2, Qwen3-14B, and Llama 3.1 as molecular-text generators, not as DDA workflow agents. The framework ablation fixes Qwen3-14 B through Ollama as the agent-generation backend, while GPT-5.2 is used only as a temperature-0 rubric judge for planning and recovery outputs. These experiment-specific settings are reported separately to avoid conflating the production DDA workflow model with direct-generation baselines or ablation backends.

The condition denoted as DDA w/o center-guided localization is a control in which the structural-context preparation step of DDA is bypassed, and TamGen receives all polymer residues as input without any center-derived local cropping. Unlike DDA and all other baselines, this condition does not use a structural center derived from the reference ligand or from any tool-based detection. It is included to quantify the contribution of the framework’s structural-context preparation module.

### 4.4 Evaluation metrics

We report molecular quality and computational prioritization metrics, including QED, synthetic accessibility (SA), Vina-family docking-oriented score, molecular diversity, and a joint screen-pass rate. The joint screen-pass rate is defined as the proportion of molecules simultaneously satisfying QED >0.5 , SA <4.0 , and Vina-family score <−5.0 . These thresholds define a fixed multi-objective computational triage policy rather than universal drug-development cutoffs. QED above 0.5 indicates at least moderate drug-likeness under the QED heuristic (Bickerton et al., 2012); SA below 4.0 excludes candidates with relatively high estimated synthetic complexity under the SA score (Ertl and Schuffenhauer, 2009); and a Vina-family score below −5.0 serves as a permissive docking-oriented screen under the fixed receptor-preparation and Uni-Dock protocol (Trott and Olson, 2010; Yu et al., 2023). The joint metric is intended to avoid optimizing a single descriptor in isolation. All individual descriptors are also reported. Together, these computational signals support candidate prioritization and provide a starting point for subsequent experimental validation, rather than replacing direct biochemical or pharmacological measurements.

### 4.5 Main comparison with baselines

We compare DDA with three complementary categories of baselines. First, specialized SBDD generators characterize molecular-output profiles under their reported generation protocols. Second, fresh matched raw-20 controls characterize the practical generation and downstream-processing behavior of the integrated TamGen pathway with and without reference-center-guided local residue selection. Third, direct target-informed text-to-SMILES baselines test whether general LLMs can provide an equivalent downstream delivery chain when they receive standardized textual target context but do not invoke the DDA workflow or a native pocket-conditioned molecular generator.

**FIGURE 3:**
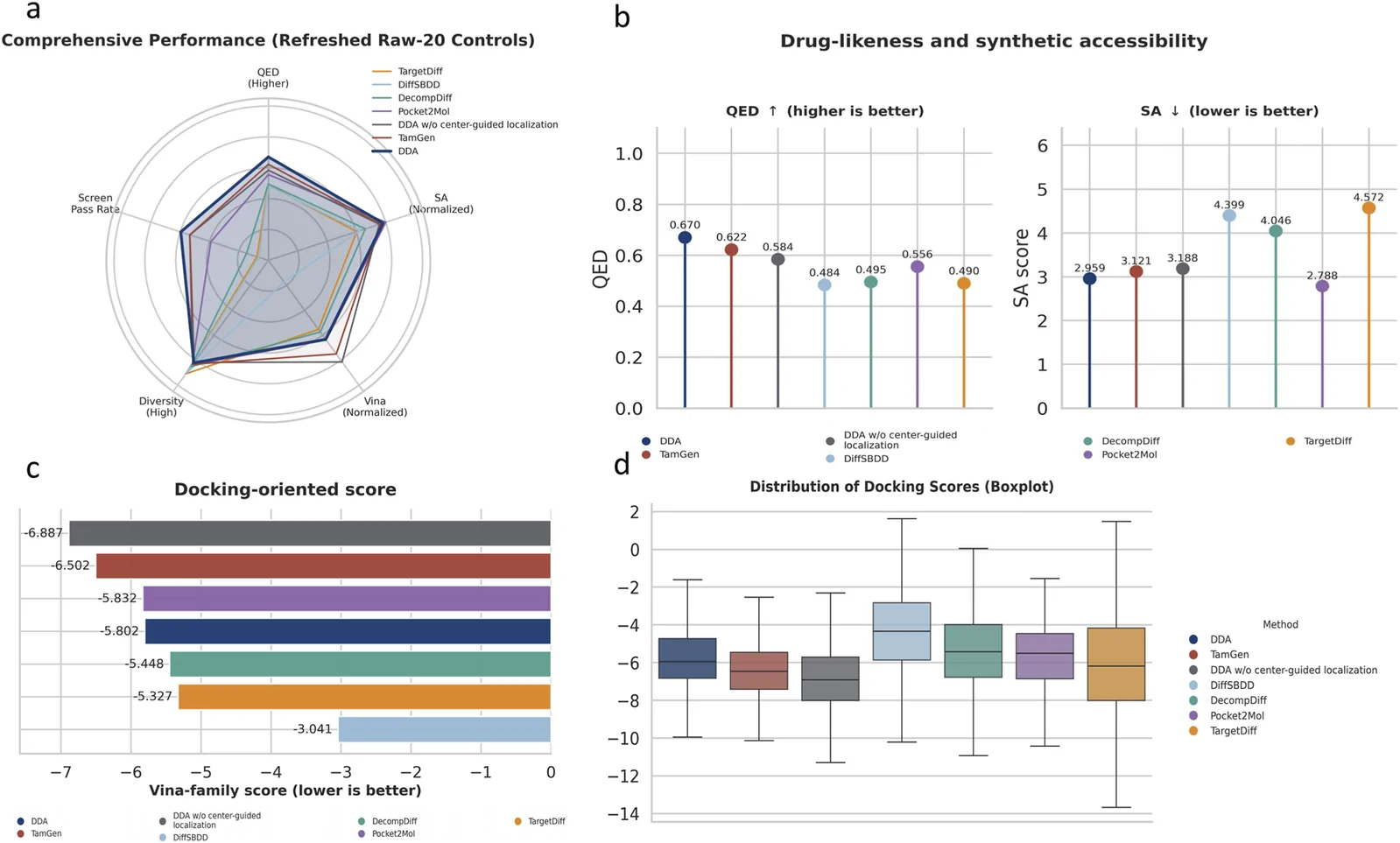
Comparison of DDA, fresh matched TamGen controls, and specialized structure-based molecular-generation baselines on CrossDocked 2020. **(A)** is a radar plot summarizing QED, normalized SA, normalized Vina-family score, diversity M , and the joint screen-pass rate. **(B)** compares mean QED and SA values. **(C)** compares mean Vina-family scores. **(D)** shows molecule-level Vina-family score distributions.

Table 3 summarizes the molecular-output profiles of DDA, the fresh TamGen controls, and specialized SBDD baselines. All methods are evaluated in the 100-target CrossDocked2020 test setting, for which the intended delivery target is 20 candidate molecules per target, corresponding to 2,000 requested candidates. The reported molecule count indicates the number of molecule-level records available in the corresponding final result file. This distinction makes delivery completeness visible without conflating the common experimental target with the observed output count. DDA is reported under its closed-loop final-delivery protocol, whereas the two fresh TamGen controls use a fixed raw-20 request and retain their observed final molecule counts without equal-count supplementation. Diversity M in Table three is the molecular diversity metric used in the radar visualization of Figure 3. Accordingly, the table compares molecular-output profiles and supports the trade-offs visualized in Figure 3. It is not interpreted as a matched same-budget intrinsic molecular-quality comparison between DDA final delivery and the raw-20 TamGen controls.

**TABLE 3:** Molecular-output profiles of DDA, fresh matched TamGen controls, and specialized SBDD baselines on the 100-target CrossDocked2020 test setting. Each condition has an intended delivery target of 20 candidates per target (2,000 requested candidates).

Method	Requested candidates	Reported molecules	QED ↑	SA ↓	Vina ↓	Diversity M ↑	Screen pass ↑
DiffSBDD	2,000	1,963	0.484	4.399	−3.041	0.904	0.065
DecompDiff	2,000	1,651	0.495	4.046	−5.448	0.852	0.148
Pocket2Mol	2,000	2,777	0.556	2.788	−5.832	0.838	0.392
TargetDiff	2,000	1,814	0.490	4.572	−5.327	0.909	0.078
TamGen	2,000	1,589	0.622	3.121	−6.502	0.833	0.536
DDA	2,000	2,000	0.670	2.959	−5.802	0.822	0.597
DDA (w/o center-guided)	2,000	1,103	0.584	3.188	−6.887	0.819	0.535

DDA produced 2,000 final candidate records across 100 targets under the predefined closed-loop delivery protocol. All records contained the required downstream fields, including ADMET-oriented evaluation results and valid docking-oriented scores. At the molecule level, 59.7% of DDA-delivered candidates passed the full multi-objective screen (QED > 0.5, SA < 4.0, and Vina < -5.0), compared with 39.2% for the best-performing specialized baseline (Pocket2Mol) and 7.8% for TargetDiff. This corresponds to an absolute improvement of approximately 20 percentage points over the strongest reported baseline. This improvement is primarily attributed to the closed-loop delivery protocol, which combines task-conditioned planning, multi-stage validation, and bounded recovery to maintain both candidate quantity and quality through the downstream evaluation chain.

The fresh raw-20 controls are designed to isolate the contribution of the framework’s structural-context preparation step. All baselines in Table three (DiffSBDD, DecompDiff, Pocket2Mol, TargetDiff, and the pocket-localized TamGen control) derive their structural center from the reference-ligand coordinates provided in the CrossDocked2020 data. This is also true for the complete DDA workflow, which obtains the structural center through tool-based detection (e.g., Fpocket) or user-provided input. In contrast, the DDA w/o center-guided localization control bypasses this preparation step entirely, supplying all polymer residues directly to TamGen without any center-derived local cropping. The center-localized TamGen condition produced 1,589 downstream-processable molecules across 100 targets, whereas the center-free control produced only 1,103 molecules, with lower mean QED (0.622 vs. 0.584) and lower screen-pass rate (0.536 vs. 0.535). These results confirm that providing a defined structural center and local protein environment substantially improves both the practical yield and molecular quality of generated candidates. Importantly, in realistic deployment scenarios where a reference ligand is not directly available, DDA invokes tools such as Fpocket to determine a binding-site center, achieving generative quality comparable to the center-informed TamGen control. The DDA w/o center-guided condition thus represents the lower-bound performance when the framework’s context-preparation tools are bypassed, while DDA leverages these tools to match or exceed the performance of center-informed baselines.

The specialized SBDD baselines provide complementary molecular-output profiles. DDA has the highest mean QED among the reported methods and a favorable joint screen-pass rate under the fixed operational thresholds. However, no method dominates every individual descriptor. For example, the raw-20 controls show lower mean Vina-family values, while Pocket2Mol has lower mean SA. These results support a workflow-level interpretation of DDA. Rather than modifying the underlying generative model, DDA automates the tedious cycle of generation, filtering, and regeneration that would otherwise require manual intervention. The framework’s closed-loop delivery protocol ensures that users receive a specified number of candidates that have passed multi-objective screening, rather than a large pool of unfiltered outputs. This is reflected in the delivery completeness of DDA (2,000/2,000) compared with the raw-20 TamGen control (1,589/2,000). The contribution of DDA thus lies not in replacing the molecular generator, but in relieving human experts from repetitive candidate triage and delivering usable, prioritized molecules ready for downstream consideration.

The direct LLM comparison is reported separately in Table 4. GPT-5.2, Qwen3-14B, and Llama 3.1 are evaluated as direct target-informed text-to-SMILES generators. For each CrossDocked2020 test target, the models receive a standardized textual context containing the target identifier and test index, canonical reference-ligand SMILES, ligand descriptors, ligand-derived center coordinates, and a summary derived from the local protein structure. In the few-shot setting, all models additionally receive the same three demonstrations selected exclusively from the CrossDocked2020 training split. These models do not invoke DDA modules, do not receive the native spatial pocket representation used by TamGen, and do not perform DDA-style tool-grounded execution or recovery.

**TABLE 4:** Direct target-informed text-to-SMILES baselines under the few-shot protocol on the 100-target CrossDocked2020 test setting. Each model is requested to provide 20 candidate molecules per target (2,000 requested candidates).

Model	Requested candidates	Valid SMILES	ADMET available	Docking valid	QED ↑	SA ↓	Vina subset mean ↓
GPT-5.2	2,000	2,000	2,000	96	0.554	3.020	−6.954
Qwen3-14 B	2,000	2,000	2,000	98	0.631	2.931	−6.961
Llama 3.1	2,000	2,000	2,000	98	0.675	2.956	−7.050

The direct-LLM experiments test whether general-purpose language models can substitute for the DDA workflow through few-shot prompting alone. Under this protocol, each model is instructed to generate 20 SMILES strings per target using the same textual target context provided to DDA, but without access to the native 3D pocket representation, the TamGen molecular generator, or any DDA tool modules. All three models produced 2,000 valid and ADMET-available SMILES after supplementation, but fewer than 100 molecules per model—less than 5% of the requested total—yielded valid docking scores. This sharp drop-off at the docking stage reveals a fundamental limitation of direct LLM-based generation: while these models can produce syntactically valid molecular strings, they lack the spatial and structural awareness required to generate molecules that are compatible with a specific binding site. In contrast, DDA integrates a structure-conditioned generator (TamGen) with tool-grounded execution and multi-stage validation, producing 2,000 fully docking-valid candidates that pass the same downstream evaluation chain. These results confirm that general-purpose LLM prompting alone is not a viable substitute for a tool-integrated, structure-aware SBDD workflow.

The direct-LLM experiments therefore do not test whether an unconditioned model can replace DDA. Instead, they test whether direct target-informed text prompting can substitute for a structure-conditioned, tool-integrated, state-aware workflow. Under the reported protocol, direct prompting can produce valid molecular text after supplementation, but it does not provide native pocket-conditioned generation, deterministic artifact handling, downstream delivery validation, or bounded recovery.

### 4.6 Comparison with specialized SBDD baselines

To further illustrate the molecular-output profiles summarized in Table three, Figure 3 compares DDA with fresh matched TamGen controls and specialized structure-based molecular-generation baselines from five complementary perspectives: QED, normalized SA, normalized Vina-family score, diversity M , and the joint screen-pass rate. The figure also shows mean QED and SA values, mean Vina-family scores, and molecule-level Vina-family score distributions. This visualization is intended to make the trade-offs among candidate quality, diversity, docking-oriented evaluation, and output-delivery behavior explicit rather than to rank all methods by a single metric.

The fresh matched controls are designed to quantify the contribution of the framework’s structural-context preparation step. All baselines reported in Table three derive their structural center from the reference-ligand coordinates in the CrossDocked2020 data—this applies to DiffSBDD, DecompDiff, Pocket2Mol, TargetDiff, and the pocket-localized TamGen control, as well as to the complete DDA workflow. The DDA w/o center-guided localization control is the only condition that does not use any structural center; it bypasses the framework’s context-preparation step and supplies all polymer residues directly to TamGen. By comparing this center-free condition with TamGen (which uses the reference-ligand center) and with DDA (which obtains a center via framework tools), this control isolates the contribution of structural-context preparation. Unlike DDA final delivery, neither control applies equal-count supplementation; partial target outputs are retained as observations of actual generation behavior under the fixed raw-20 configuration.

The DDA w/o center-guided localization control allows us to assess the value of the framework’s structural-context preparation tools. In this condition, TamGen receives the full protein sequence without any center-derived local cropping, whereas the pocket-localized TamGen control uses the reference-ligand-derived center to select local residues. The center-localized condition produced substantially more downstream-processable molecules (1,589 vs. 1,103 across 100 targets) and achieved higher QED (0.622 vs. 0.584) and screen-pass rate (0.536 vs. 0.535). These results confirm that providing a defined structural center is critical for both the yield and quality of generated candidates. Importantly, in realistic deployment scenarios where a reference ligand is not directly available, DDA invokes tools such as Fpocket to determine a binding-site center, achieving generative quality comparable to the center-informed TamGen control. This control thus validates the practical utility of the framework’s tool-integrated structural-context preparation, independent of the specific method used to obtain the center.

DDA produced 2,000 final candidate records under its closed-loop delivery protocol and showed the highest mean QED among the methods reported in Table three. It also maintained favorable SA and a joint screen-pass rate of 0.597 under the predefined computational triage thresholds. However,Figure 3 also shows that DDA does not dominate every individual molecular descriptor. The fresh raw-20 TamGen controls show lower mean Vina-family scores, Pocket2Mol shows a lower mean SA value, and several specialized generators exhibit higher diversity values. These observations are important because they distinguish the contribution of DDA from a claim of universal single-metric superiority.

Overall, these results support DDA workflow interpretation. First, DDA’s closed-loop delivery protocol combines bounded supplementation, deterministic product validation, and multi-objective screening, achieving a high screening pass rate. This improvement reflects DDA’s ability to automate generation, filtering, and regeneration iteration cycles, providing a specified number of available candidate products instead of a large number of unfiltered outputs. Second, comparing TamGen with the centerless guided positioning DDA confirmed that the framework’s structural-contextual preparation tools are crucial for maintaining candidate yield and quality in real-world scenarios without reference ligands. These results collectively support a workflow-level interpretation of DDA: its value lies not in improving the underlying generator, but in reducing the redundant candidate screening of human experts through closed-loop delivery. The following ablation analysis further analyzes which components—planning, tool selection, code execution, and memory I/O—contribute to the execution of this reliable workflow.

The comparison also clarifies the role of the additional controls in addressing the generator-related confounding factor. The fresh pocket-localized control provides a direct raw-20 reference for the integrated target-aware generator, and the DDA w/o center-guided localization condition isolates the implemented structural-context component. Together with the framework ablations described below, these results separate three complementary questions: how the generation pathway behaves under fixed controls, how DDA delivers final candidate products through a closed-loop workflow, and how individual agentic components contribute to task-conditioned workflow execution.

### 4.7 Ablation analysis

The molecular-output experiments above evaluate candidate products and closed-loop delivery, but they do not by themselves establish which framework components support task-conditioned workflow execution. We therefore conducted a set of controlled module-isolation experiments. These experiments are not a molecular-quality benchmark and do not reuse the 2,000-candidate CrossDocked2020 delivery setting. Instead, they evaluate four complementary framework functions: initial workflow planning, registry-grounded tool selection, executable-wrapper generation and validation, and state-aware recovery planning.

These subexperiments use distinct controlled task suites and evaluation procedures. Qwen3-14 B through Ollama is fixed as the generation backend for the agent variants. GPT-5.2 is used only as a temperature-0 rubric judge for planning and recovery outputs. It does not generate the plans, tool selections, or code wrappers that are evaluated. All variants within each subexperiment are evaluated on the same task records. The records are controlled workflow-state templates with repeated instances, and the reported means and standard deviations are descriptive summaries rather than population-level significance estimates.

The “Real execution” column in Table 5indicates whether the subexperiment involves actual runtime execution of generated code (Code execution) or relies solely on static plan or selection quality assessments (Planning, Tool selection, Recovery).

**TABLE 5:** Framework-ablation protocol for the four controlled subexperiments. The four subexperiments evaluate distinct framework functions and do not estimate one common end-to-end success rate.

Subexperiment	Records	Evaluation source	Real execution
Planning	100	GPT-5.2 fixed-rubric assessment of raw plan JSON	No
Tool selection	120	Deterministic comparison with task-specific gold tool sets	No
Code execution	80	Generated wrappers, subprocess execution, and deterministic artifact checks	Yes
Recovery	60	GPT-5.2 fixed-rubric assessment of recovery plans	No

For planning, each task defines a natural-language request, declared available state, required capabilities, expected stage order, and prohibited actions. The full Planner is compared with a static generic workflow template and a ‘w/o Planner’ direct-step condition. Planning outputs are assessed for stage coverage, stage-order alignment, forbidden actions, and executability. These are continuous plan-alignment measures rather than binary end-to-end execution rates. The full Planner achieves stage coverage of 0.691, stage-order alignment of 0.643, a forbidden-action rate of 0.049, and executability of 0.632. The static template achieves lower coverage (0.535), lower order alignment (0.399), a substantially higher forbidden-action rate (0.420), and lower executability (0.300). The ‘w/o Planner’ condition has low coverage (0.171), order alignment (0.060), and executability (0.069). Its low forbidden-action value reflects under-specification rather than successful task adaptation: a plan with few actions can avoid prohibited actions while failing to cover the required workflow.

Tool selection is evaluated by comparing the selected tool set S with a task-specific gold tool set G . We report precision, recall, and F1 as defined in Equation 12:

$$Precision=\frac{\left|S\cap G\right|}{\left|S\right|},\;Recall=\frac{\left|S\cap G\right|}{\left|G\right|},F1=\frac{2\cdot Precision\cdot Recall}{Precision+Recall}.$$ (12)

We additionally report the over-selection penalty, |S\G|/|S| , with an empty selected set assigned a penalty of zero, and critical-tool miss, which is one when at least one gold tool is absent from S. The full ToolSelector achieves the highest precision (0.953) and F1-score (0.756), while retaining a low over-selection penalty (0.047). The rule-based selector has higher recall (0.764) than the full ToolSelector but lower precision (0.664) and F1-score (0.666). The “w/o Tool Selector” condition exposes all available tools without selective tool grounding. It achieves recall of 1.000 but precision of only 0.323, an F1-score of 0.484, and an over-selection penalty of 0.677. Thus, maximal tool exposure is not equivalent to task-relevant tool grounding.

The code-execution suite evaluates the generation and execution of lightweight scientific wrappers using local inputs, declared expected outputs, and required CSV columns or JSON keys. The full condition includes tool context, evaluator feedback, bounded refinement, execution, and memory I/O. The ablations remove one specified component while retaining the controlled task specification. Reported outcomes include generated-wrapper availability, evaluator acceptance, subprocess execution success, expected-output existence, output-schema validity, and validated output correctness. Semantic correctness is based on expected artifact existence, parseability, required schema fields, and non-empty outputs. It is a task-level output-validation measure and is not a claim of deep chemical or biological semantic validation.

The full code-execution condition achieves execution success of 0.800, output-schema validity of 0.575, and validated output correctness of 0.562. Removing the Evaluator reduces validated output correctness to 0.450, while ‘w/o Refinement’ reduces it to 0.475. Removing tool context reduces execution success to 0.662 and validated output correctness to 0.375. The largest degradation occurs for ‘w/o Memory I/O’, where execution success falls to 0.088, output-schema validity to 0.050, and validated output correctness to 0.050. These results indicate that explicit input-output state transfer is important for producing usable local workflow products. The current generated-wrapper availability metric is reported as such. It should not be interpreted as an independent Python compilation rate.

The recovery suite evaluates proposed recovery actions for declared interrupted workflow states. Its rubric considers whether a recovery plan selects appropriate actions, avoids redundant work, avoids wrong-stage actions, predicts output recovery, and identifies the need for manual intervention. Full memory achieves an avoided-redundant-work rate of 0.505 and predicted recovery success of 0.367, compared with 0.025 and 0.167, respectively, for ‘w/o Memory’. These recovery outcomes are assessments of recovery-plan quality under controlled declared states, not physical re-execution of every full molecular workflow.

**FIGURE 4:**
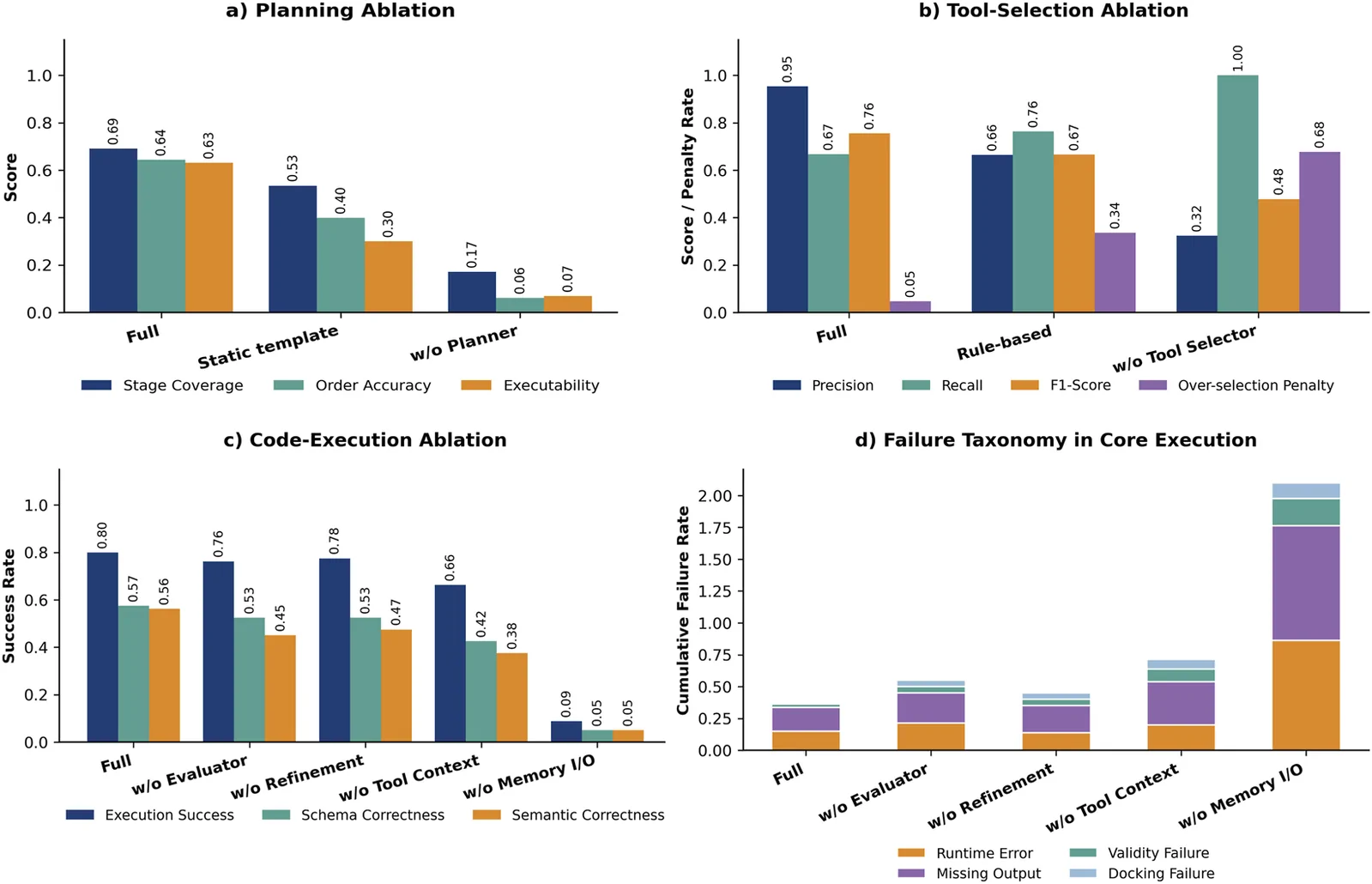
Framework ablation results. **(A)** planning ablation comparing Full, Static template, and w/o Planner. **(B)** tool-selection ablation comparing Full, Rule-based, and w/o Tool Selector. **(C)** code-execution ablation comparing Full with w/o Evaluator, w/o Refinement, w/o Tool Context, and w/o Memory I/O. **(D)** diagnostic failure taxonomy.

Figure 4 visualizes the principal planning, tool-selection, and code-execution ablations. Panel (A) shows that the full Planner provides more complete and better ordered initial plans than a static full-workflow template or a direct no-planner condition. Panel (B) shows the precision–recall trade-off in tool selection: the all-tools strategy obtains complete recall by exposing every tool but incurs substantial over-selection, whereas the full ToolSelector obtains the strongest precision and F1. Panel (C) shows that tool context, evaluator/refinement support, and especially memory I/O contribute to executable and validated outputs. Panel (D) provides a diagnostic failure taxonomy from the available execution summary. It is interpreted descriptively as a breakdown of recorded runtime, missing-output, validity, and docking-related failures, and is not used as a direct replacement for the controlled code-execution metrics above.

Taken together, these ablation results support a bounded architectural conclusion. The agentic components do not replace the domain-expert-defined scientific scaffold or deterministic scientific tools. Instead, within the supported workflow space, task-conditioned planning improves initial plan alignment relative to a generic static template, selective tool grounding improves precision relative to indiscriminate all-tool exposure, evaluation and refinement improve wrapper-level output checks, and explicit memory I/O supports cross-step artifact delivery. These component-level results complement, rather than duplicate, the closed-loop molecular-delivery evidence reported in the main comparison.

### 4.8 Web-based interactive execution and audit interface

**FIGURE 5:**
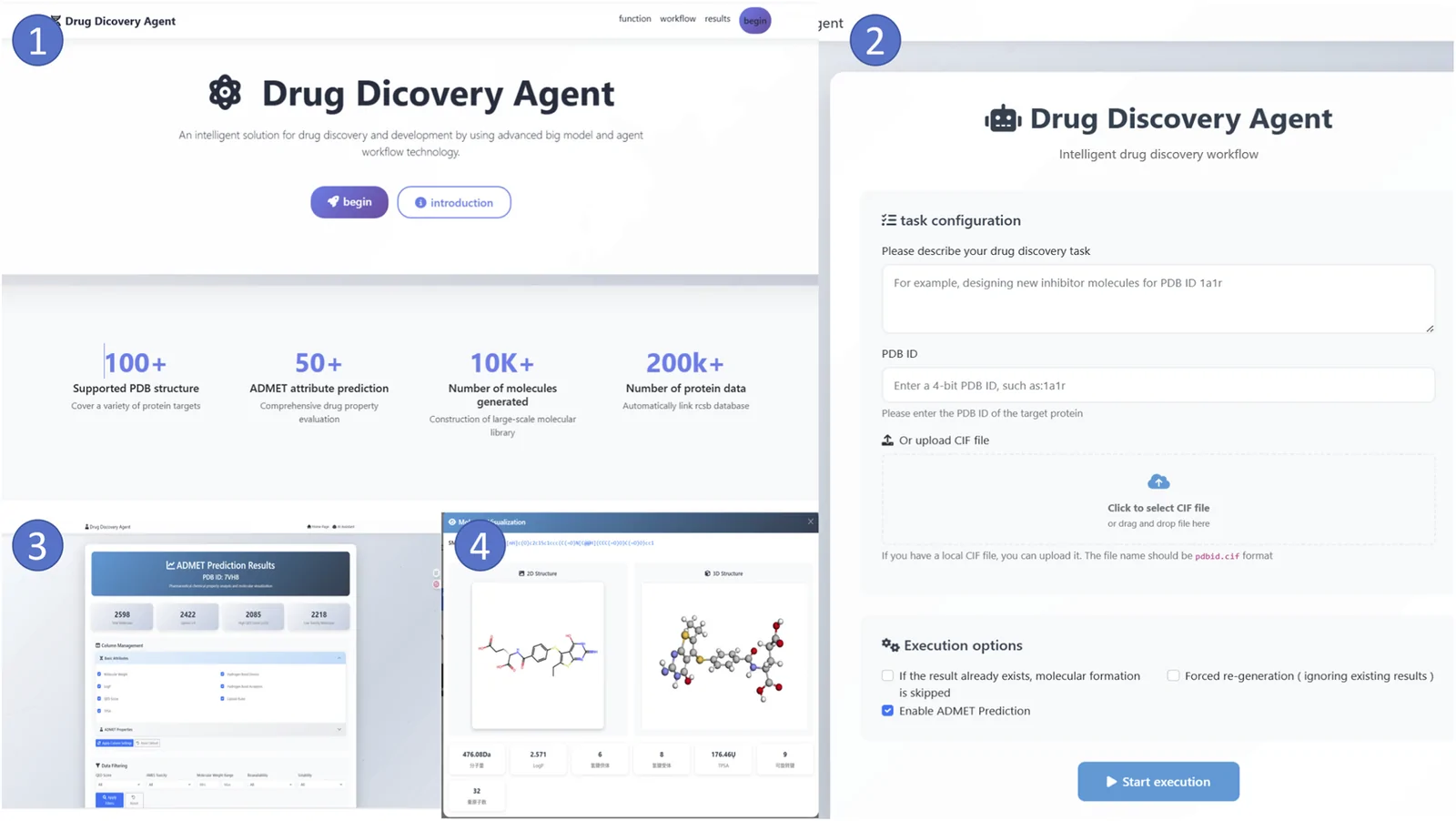
Web-based execution and audit interface of DDA. (1) shows the main entry page. (2) shows the task configuration form, which accepts a natural-language request, PDB identifier, optional CIF file, candidate count, center mode, and fresh-run option. (3) shows the filterable result table with molecular, ADMET, docking, and ranking fields. (4) shows the 2D and 3D molecular inspection views.

DDA provides a Web application and command-line interface as execution and audit surfaces rather than as post hoc visualization layers. As illustrated in panels (1) and (2) of Figure 5, the Web interface accepts a natural-language drug-design request and a PDB identifier, and optionally accepts a local CIF structure file, requested final-candidate count, structural-center mode, explicit center coordinates, and a forced fresh-run option. Submitting the form launches the operational workflow rather than a display-only preview. The runtime then initializes GlobalMemory, records the task configuration, checks available products, invokes product-aware planning where appropriate, and coordinates tool selection, wrapper evaluation, execution, and final result validation. During execution, the interface exposes job status, stage progress, recorded agent activity, execution messages, output locations, and final delivery status.

After workflow completion, the result interface presents a filterable candidate table with molecular descriptors, ADMET-related properties, docking-oriented scores, and ranking fields, as shown in panel (3) of Figure 5. Users can select individual candidate molecules for two-dimensional and three-dimensional structural inspection, as illustrated in panel (4). Through these interfaces, DDA provides users with direct access to workflow configuration, execution state, intermediate products, and candidate review, thereby enabling informed decision making at each stage of the design process.

## 5 End-to-end workflow case study

To further demonstrate the practical task-completion capability of DDA, we conduct an end-to-end workflow case study on CrossDocked2020 target 3daf. Different from the quantitative comparisons and module-level ablation experiments, this case study focuses on whether DDA can transform a natural-language drug-design request into a complete, executable, inspectable, and recorded SBDD workflow for a real target. The objective is not to establish generalization from a single target, but to provide a concrete demonstration of the framework behavior that underlies the aggregate workflow-delivery results.

The input is a natural-language request asking the system to generate 20 candidate drug molecules for target 3daf. After receiving the task, the Planner converts the request into a structured execution plan. The plan covers target preparation, structural-context and seed setup, molecular generation, post-processing, ADMET-oriented evaluation, final candidate selection, docking, docking-score write-back, and result reporting. This plan defines the stage dependencies and execution order for the downstream agents.

After planning, DDA enters the agent-supported execution loop. For each step, the ToolSelector identifies the relevant registered tools, the CodeProgrammer generates the corresponding executable wrapper, the Evaluator checks the input-output paths and execution logic, and the Executor runs the tool invocation and writes the resulting products back to GlobalMemory. GlobalMemory maintains target information, structural-context information, intermediate files, execution records, and result tables across the workflow, allowing later stages to locate upstream products and continue without losing execution state.

**FIGURE 6:**
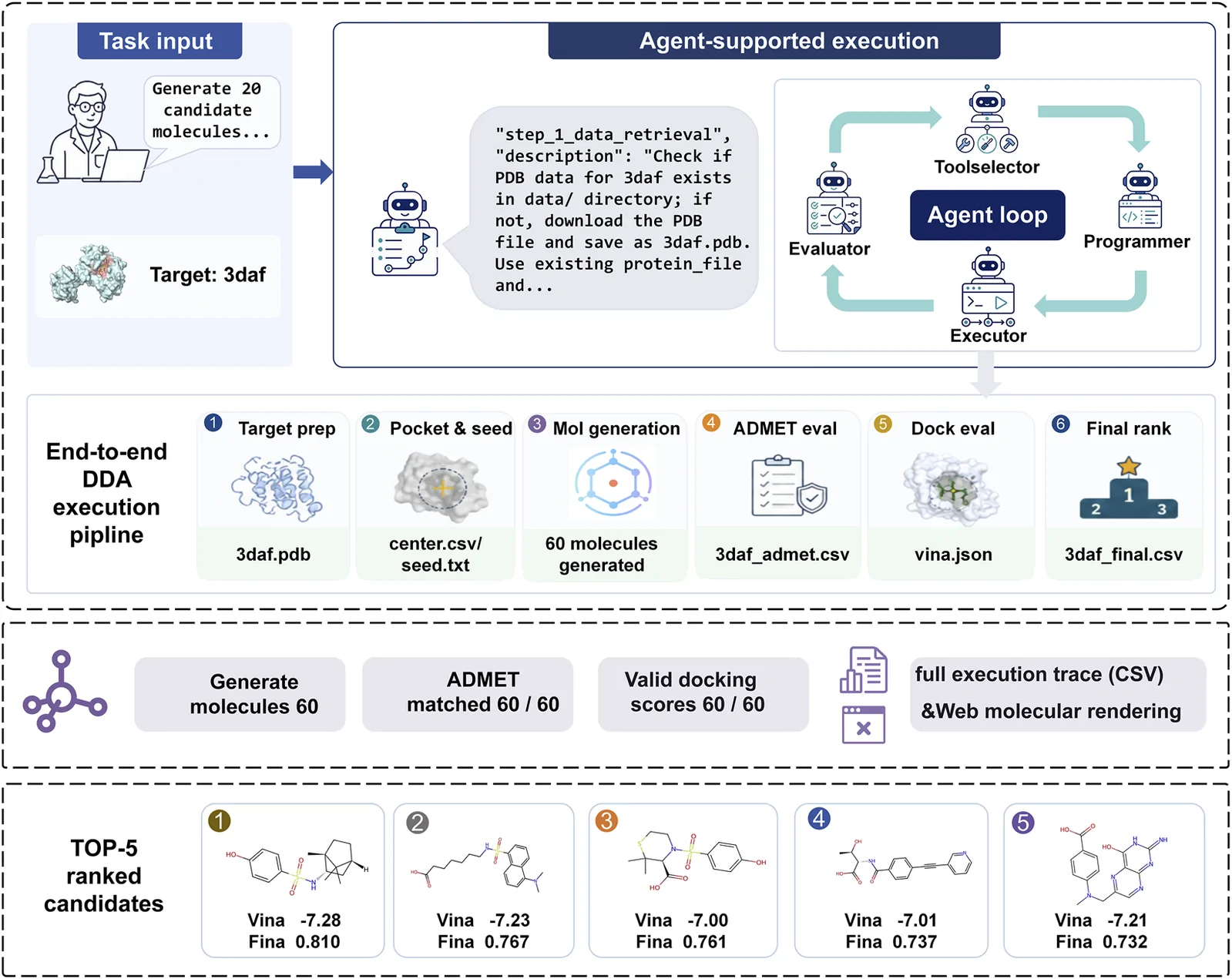
End-to-end workflow case study on target 3daf. The figure shows the complete execution pipeline, including the structured workflow plan, the six functional stages, and the Top-5 ranked candidates. The 3daf case generates 60 molecules with 60/60 ADMET-matched outputs and 60/60 valid docking-oriented scores, and returns a Top-20 ranked set with a full execution trace and visualization outputs.

Figure 6 shows the complete execution process for target 3daf. DDA first prepares the target structure and determines the structural center and seed molecules from the reference ligand, producing the center and seed products required for molecular generation. The system then generates 60 candidate molecules. After post-processing, all 60 molecules enter ADMET evaluation, producing 60/60 ADMET-matched outputs. Docking evaluation subsequently obtains 60/60 valid docking-oriented scores and writes these values into the candidate result records. The final ranking stage integrates the available molecular properties, ADMET-related fields, and docking-oriented scores, and returns the Top-20 candidate set requested by the user.

The complete execution trace for this target records the task request, generated workflow plan, selected tools, executable wrappers, evaluator feedback, execution messages, intermediate output files, ADMET results, docking results, and final candidate table. The resulting files include the prepared structural-center and seed products, the ADMET result table, docking-oriented output records, final ranked candidate records, and web-based molecular visualization results. Thus, the case demonstrates that DDA produces not only a final molecule list, but also the computational evidence needed to inspect how the final candidates were generated, processed, evaluated, and ranked.

The Top-5 candidates shown in Figure 6 illustrate the final result representation available to users. Rank 1 has a Vina-family score of −7.28 and a final score of 0.810; Rank 2 has a Vina-family score of −7.23 and a final score of 0.767; Rank 3 has a Vina-family score of −7.00 and a final score of 0.761; Rank 4 has a Vina-family score of −7.01 and a final score of 0.737; and Rank 5 has a Vina-family score of −7.21 and a final score of 0.732. These values are computational prioritization outputs under the configured workflow, intended to support candidate comparison and inspection. They serve as a starting point for further experimental validation rather than as direct estimates of binding affinity or biological activity.

This case study demonstrates the practical value of combining LLM-based task interpretation with tool-grounded execution and GlobalMemory-based state transfer. The workflow connects a user request to target-specific candidate generation, standardized downstream evaluation, ranked output, and human-facing molecular inspection without requiring researchers to manually coordinate every intermediate software invocation and file transfer. In a practical drug-discovery setting, such a workflow can support medicinal-chemistry review, synthesis or procurement prioritization, and subsequent experimental testing. The 3daf case provides a representative end-to-end demonstration, while the 100-target CrossDocked2020 benchmark and Ablation Analysis module-isolation experiments provide the corresponding aggregate molecular-delivery and framework-mechanism evidence.

## 6 Discussion

The results position DDA as a workflow-level biomedical informatics framework rather than as a replacement for specialized molecular generators, ADMET predictors, docking engines, or medicinal-chemistry expertise. Its main contribution is to connect local target preparation, structure-conditioned generation, molecular processing, ADMET-oriented prioritization, final-candidate docking-score write-back, product validation, and result inspection into one task-conditioned workflow. DDA therefore addresses a practical coordination problem in SBDD: researchers often need to manage heterogeneous software, intermediate files, parameters, and downstream evaluation steps before a generated molecule can become an inspectable candidate record.

The molecular-output results should be interpreted in this workflow context. DDA delivers 2,000 final candidate records under the closed-loop delivery protocol, whereas the fresh raw-20 TamGen controls report the observed outputs of a fixed generation configuration without equal-count supplementation. The pocket-localized TamGen control produces more downstream processable molecules than the condition without center-guided localization, supporting the value of reference-center-guided local residue selection in the integrated TamGen pathway. At the same time, DDA does not dominate every individual molecular descriptor: the raw-20 controls show lower mean Vina-family values, Pocket2Mol has lower mean SA, and several specialized generators show higher diversity. Thus, the contribution of DDA is not universal single-metric superiority over TamGen or every specialized SBDD baseline. Rather, it is the delivery of a ranked, multi-objectively prioritized, and auditable candidate set through an integrated workflow.

The Ablation Analysis results provide complementary evidence for the agentic architecture. Planning, tool selection, code execution, and recovery are evaluated on separate controlled task suites and do not estimate the same quantity as the 100-target closed-loop delivery benchmark. Within the supported workflow space, task-conditioned planning improves initial plan alignment relative to a static generic template, selective tool grounding improves precision relative to indiscriminate all-tool exposure, and explicit memory I/O supports executable artifact delivery. These findings do not imply that an LLM freely discovers scientific procedures or is necessary for every fixed workflow branch. Instead, DDA uses a domain-expert-defined scaffold in which LLM components adapt supported workflow paths according to user requests, available products, local inputs, and runtime state, while deterministic tools perform scientific computation and validation.

ChemCrow provides an important framework-level reference because it establishes the general paradigm of combining LLM reasoning with expert chemistry tools. DDA extends this direction toward local structure-conditioned molecular-design workflow delivery, including structural-context preparation, target-aware TamGen generation, ADMET-oriented prioritization, final-candidate docking, runtime product management, bounded recovery, and Web/CLI execution visibility. The current ChemCrow comparison is therefore capability-level rather than numerical: the systems do not share an identical tool registry, structural representation, molecular generator, output contract, or runtime budget. A same-tool generic-agent baseline using the DDA registry, local inputs, output contract, and timeout policy will be an important future experiment for directly isolating workflow-architecture effects.

DDA has immediate practical value as a computational triage system that supports human oversight and intervention. It can reduce repeated manual coordination across structural preparation, candidate generation, property evaluation, docking, result aggregation, and record preservation, while providing medicinal chemists with a candidate set that can be inspected and prioritized according to project-specific constraints. The current framework can support target-focused virtual screening, lead identification, synthesis or procurement prioritization, and subsequent experimental review. Future extensions can integrate experimental agents for synthesis planning, assay selection, laboratory information management, experimental-result ingestion, and active-learning updates, thereby connecting the present computational workflow to a design-make-test-learn cycle. Nevertheless, the current benchmark uses reference-ligand-derived centers and computational proxy metrics. QED, SA, predicted ADMET, diversity, and Vina-family values support candidate prioritization, but experimental binding, activity, selectivity, pharmacokinetic, and safety studies remain necessary before biological or clinical conclusions can be drawn.

## 7 Conclusion

Automated structure-based drug design has advanced rapidly with the development of molecular generators, ADMET prediction tools, docking engines, and LLM-based agents. However, these capabilities are often used as isolated stages and require researchers to manually coordinate structural inputs, molecular files, evaluation outputs, and candidate-ranking procedures. In this study, we presented DDA, a multi-agent biomedical informatics framework that connects natural-language task requests with local target preparation, structure-conditioned molecular generation, ADMET-oriented prioritization, docking-score write-back, candidate ranking, product validation, and visualization. Rather than replacing specialized scientific tools, DDA organizes them within a domain-expert-defined, task-conditioned, and auditable workflow.

Experiments on the 100-target CrossDocked2020 setting demonstrate that DDA can provide validated closed-loop final candidate delivery under the reported computational protocol. The fresh raw-20 TamGen controls further show that reference-center-guided local residue selection affects practical downstream molecule delivery in the integrated generation pathway. The framework ablations provide complementary evidence that planning, selective tool grounding, wrapper evaluation/refinement, and explicit memory I/O support task-conditioned workflow execution. Together, these results support DDA as a workflow-delivery framework that produces ranked candidate records and associated computational products, rather than as a claim of universal molecular-property superiority over every specialized generator or direct replacement for medicinal-chemistry judgment.

Future work will extend DDA in several directions. These include incorporating flexible structural-context definition strategies, additional molecular generators and evaluation tools, synthesis-planning modules, and project-specific multi-objective ranking policies. DDA can also be extended with experimental agents for compound procurement or synthesis coordination, assay selection, laboratory-result ingestion, and iterative design-make-test-learn workflows. Prospective synthesis, biochemical binding assays, cellular activity studies, selectivity profiling, pharmacokinetic evaluation, and safety testing will be required to establish biological and translational value. In addition, a same-tool generic-agent baseline using the DDA tool registry, local inputs, output contract, and runtime policy will enable a more direct comparison of workflow architecture with general chemistry-agent frameworks. By combining constrained LLM orchestration with deterministic scientific execution and recorded workflow products, DDA provides a practical foundation for scalable, inspectable computational drug discovery that supports expert oversight and informed decision making.

## Data Availability

The original contributions presented in the study are included in the article/supplementary material, further inquiries can be directed to the corresponding author.
